# Agonist and antagonist binding to the nuclear vitamin D receptor: dynamics, mutation effects and functional implications

**DOI:** 10.1186/2193-9616-1-2

**Published:** 2013-02-12

**Authors:** Sepideh Yaghmaei, Christopher Roberts, Rizi Ai, Mathew T Mizwicki, Chia-en A Chang

**Affiliations:** 1 Department of Chemistry, University of California, Riverside, California; 2 Department of Biochemistry, University of California, Riverside, California

**Keywords:** Nuclear vitamin D receptor, Vitamin D_3_, 1α,25(OH)_2_-vitamin D_3_, Antagonist, Molecular dynamics, Helix 12, Conformational ensemble

## Abstract

**Purpose:**

The thermodynamically favored complex between the nuclear vitamin D receptor (VDR) and 1α,25(OH)_2_-vitamin D_3_ (1,25D3) triggers a shift in equilibrium to favor VDR binding to DNA, heterodimerization with the nuclear retinoid x receptor (RXR) and subsequent regulation of gene transcription. The key amino acids and structural requirements governing VDR binding to nuclear coactivators (NCoA) are well defined. Yet very little is understood about the internal changes in amino acid flexibility underpinning the control of ligand affinity, helix 12 conformation and function. Herein, we use molecular dynamics (MD) to study how the backbone and side-chain flexibility of the VDR differs when a) complexed to 1α,25(OH)_2_-vitamin D_3_ (1,25D3, agonist) and (23S),25-dehydro-1α(OH)-vitamin D_3_-26,23-lactone (MK, antagonist); b) residues that form hydrogen bonds with the C25-OH (H305 and H397) of 1,25D3 are mutated to phenylalanine; c) helix 12 conformation is changed and ligand is removed; and d) x-ray water near the C1- and C3-OH groups of 1,25D3 are present or replaced with explicit solvent.

**Methods:**

We performed molecular dynamic simulations on the apo- and holo-VDRs and used T-Analyst to monitor the changes in the backbone and side-chain flexibility of residues that form regions of the VDR ligand binding pocket (LBP), NCoA surface and control helix 12 conformation.

**Results:**

The VDR-1,25D3 and VDR-MK MD simulations demonstrate that 1,25D3 and MK induce highly similar changes in backbone and side-chain flexibility in residues that form the LBP. MK however did increase the backbone and side-chain flexibility of L404 and R274 respectively. MK also induced expansion of the VDR charge clamp (i.e. NCoA surface) and weakened the intramolecular interaction between H305---V418 (helix 12) and TYR401 (helix 11). In VDR_FF, MK induced a generally more rigid LBP and stronger interaction between F397 and F422 than 1,25D3, and reduced the flexibility of the R274 side-chain. Lastly the VDR MD simulations indicate that R274 can sample multiple conformations in the presence of ligand. When the R274 is extended, the β-OH group of 1,25D3 lies proximal to the backbone carbonyl oxygen of R274 and the side-chain forms H-bonds with hinge domain residues. This differs from the x-ray, kinked geometry, where the side-chain forms an H-bond with the 1α-OH group. Furthermore, 1,25D3, but not MK was observed to stabilize the x-ray geometry of R274 during the > 30 ns MD runs.

**Conclusions:**

The MD methodology applied herein provides an in silico foundation to be expanded upon to better understand the intrinsic flexibility of the VDR and better understand key side-chain and backbone movements involved in the bimolecular interaction between the VDR and its’ ligands.

**Electronic supplementary material:**

The online version of this article (doi:10.1186/2193-9616-1-2) contains supplementary material, which is available to authorized users.

## Background

The nuclear vitamin D receptor (VDR) is a member of the nuclear receptor (NR) transcription factor super family (Nuclear receptor nomenclature committee 1999). Based on sequence and functional homology, NR’s are partitioned into five domains (A-F), of which domain C (i.e. the DNA binding domain) contains the greatest degree of sequence homology. Domain E, the ligand binding domain (LBD), varies cross-family in sequence homology; however, the transcriptionally active conformation of NR LBDs is highly conserved (Wurtz et al 1996) and is highlighted by a closed position of the C-terminal helix of domain E (helix 12), capping the ligand binding pocket (LBP) (Renaud and Moras 2000). Some NRs have been observed to adopt the closed, active conformation in the absence of ligand (apoNR, e.g. Nurr1) (Wang et al 2003). Alternatively other NRs more absolutely require an endogenous ligand to increase the stability of the active conformer.

**Figure 1:**
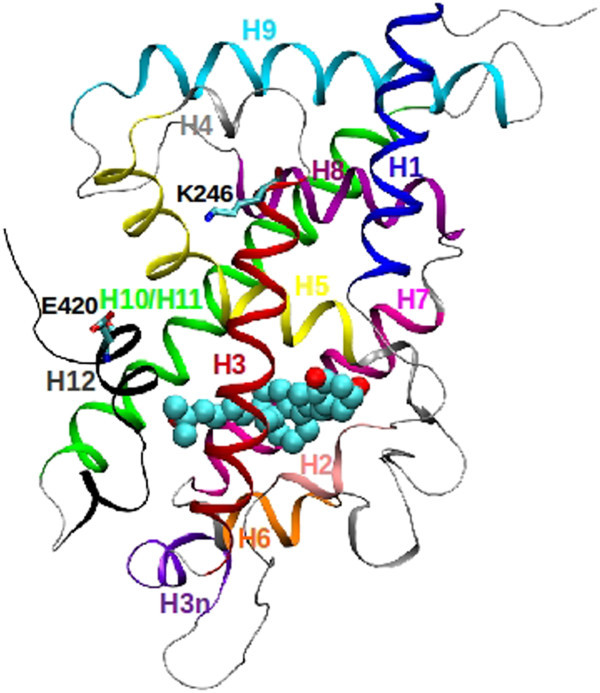
**The nuclear vitamin D receptor (VDR) bimolecular complex with 1,25D3.** The ribbon diagram of the VDR is rendered from the energy optimized complex between 1,25D3 and the VDR (aa120-427, Δ165-215), pdb code 1DB1 (Table 1). The original pdb identified 14 α-helical regions of the VDR; however, it is common practice to refer to the VDR as having thirteen α-helical regions: aa125-143 (Helix 1, H1, blue), aa149-153 (H2, pink), aa216-224 (H3n, brown), aa226-247 (H3, cyan), aa250-254 (H4, rust brown), aa255-275 (H5, lime), aa296-302 (H6, gold), aa306-323 (H7, magenta), aa326-339 (H8, purple), aa348-371 (H9, orange), aa378-406 (H10/H11, red) and aa410-423 (H12, black). The one three strand β-sheet is colored gray and consists of residues 279–294. The bound 1,25D3 ligand is shown in its’ bound, bowl-like shape, and is rendered in CPK space-filling with carbon atoms colored cyan and oxygen atoms colored red. The two residues that form the charge clamp, K246 and E420, are rendered in their tube structure with carbon atoms (cyan), oxygen atoms (red) and nitrogen atoms (blue).

NRs whose cognate ligands are cholesterol derivatives (e.g. steroids) show strong, nanomolar binding affinities. The VDR falls into this classification of NRs, given the active conformation is dramatically stabilized by binding to the seco-steroid hormone, 1α,25(OH)_2_-vitamin D_3_ (1,25D3) (Peleg et al 1995; Mizwicki et al 2009a). The VDR transcriptionally active conformation is defined by the VDR-1,25D3 x-ray co-complex (Figure 1) (Rochel et al 2000). Like the other NR family members, the closure of helix 12 completes the nuclear co-activator (NCoA) binding surface (Renaud and Moras 2000). The landscape of the NCoA binding surface for the VDR can best be described as a surface of hydrophobic residues that lie between a charge clamp that is made between a conserved LYS and GLU residue, residues 246 (helix 4) and 420 (helix 12) respectively (Figure 1).

NR ligands that sterically block helix 12 closure function as traditional NR antagonists by disrupting the formation of the NCoA surface/charge clamp. Three general classes of VDR antagonists have been created to date. The first class, based on extending the aliphatic side-chain of 1,25D3, function to sterically disrupt helix 12 closure (Carlberg 2004); the second, based on an adamantyl side-chain chemistry, more subtly disrupt helix 12 closure (Igarashi et al 2007; Nakabayashi et al 2008); and the third class, which was first to be discovered, was generated by removing the chiral end of one of the terminal side-chain metabolites of 1,25D3, to generate (23S)-1α(OH)-vitamin D_3_-26,23-lactone (MK)] (Miura et al 1999). Similar to the adamantyl-based antagonists, MK does not sterically displace helix 12 from the active position (Mizwicki et al 2009a) and does mimic 1,25D3 in its’ ability to stabilize other regions of the VDR LBD (Kakuda et al 2010; Mizwicki et al 2012). MK is a unique NR antagonist because it can be converted into a VDR superagonist (i.e. transactivation potential 10–100 fold greater than 1,25D3) by a single (H305 to F305) or double H305F/H397F (VDR_FF) point mutant (Mizwicki et al 2009a; Kakuda et al 2010).

The other extreme conformation of helix 12, with respect to the closed conformation, is the opened conformation. This conformation has been observed for a mutated and unliganded, apoRXR (Vivat et al 1997) and 17β-estradiol bound ERα (Tanenbaum et al 1998). To date, no structure has been solved that demonstrates the VDR can be stabilized in an ‘opened’ conformation, yet it is the conformation assumed for all ligand-activated apoNRs by the mouse-trap, induced-fit activation mechanism (Renaud and Moras 2000).

Solution state limited proteolytic digest of the VDR indicates that at least three distinct helix 12 conformations exist in solution (Peleg et al 1995). In specific two trypsin sites in helix 11 (R402) and helix 12 (K413), have shown differential cleavage rates when the VDR H305 and H397 residues were mutated to phenylalanine and/or following incubation with 1,25D3 or MK (Mizwicki et al 2007). More over, hydrogen-deuterium exchange coupled mass spectrometry results clearly demonstrate that different VDR agonist ligands differentially alter the flexibility of helix 12 backbone atoms (Zhang et al 2010). Thus it is clear that other techniques must be utilized besides crystallography to understand the molecular dynamics associated with the intrinsic flexibility of the VDR and its’ bimolecular interaction with ligand and how those interactions translate into the many cellular functions of active vitamin D_3_ metabolites and the VDR.

Molecular Dynamics (MD) computational simulations have been employed to observe how the population of distinct conformational states fluctuate when a protein is unliganded or complexed with agonist or antagonist ligand (Gallicchio and Levy 2011; Ai et al 2009). MD has also been used to identify novel ligand accessible binding sites in HIV integrase (Schames et al 2004). Furthermore, explicit solvent MD has been employed to demonstrate that water can play a role in allosterically regulating conformational switches (Prakash et al 2012; Gorfe and Caflisch 2005) and dictate ‘slaved’ protein motions (Fenimore et al 2002). Application of MD in the VDR field has been used to a) provide a structural basis for the species specific antagonistic nature of MK (Perakyla et al 2004); b) determine how ligand dissociates from the VDR (Perakyla 2009); and c) design novel VDR agonists (Shen et al 2012). In this work we used explicit solvent MD to assess how the backbone and side-chain flexibility of the VDR is influenced by changing helix 12 conformation, VDR primary sequence, ligand chemistry and absence of ligand.

## Methods

### Structures used for MD simulations

The initial coordinates of human Vitamin D receptor (VDR), VDR_H305F and VDR_FF (H305F/H397F double mutation) were obtained from the VDR-1α,25(OH)_2_D_3_ (1,25D3) x-ray crystal complex (PDB: 1DB1), VDR_H305F-MK complex (PDB: 3A2I) and the VDR_FF-MK complex (PDB: 3A2J) respectively. The three missing residues for the loop between helices 8 and 9 were added to the X-ray structure of VDR-LBD-1,25D3 using the loop refinement module of Discovery Studio 2.0 (Accelyrs Inc., San Diego). The initial structure of the VDR_H305F-1,25D3 and VDR_FF-1,25D3 complexes were constructed by aligning their x-ray coordinate to the minimized structure of VDR-1,25D3 complex and replacing MK with 1,25D3. The initial coordinates of VDR_H397F-1,25D3 were obtained by mutating the HIS397 residue, of the energy minimized VDR-1,25D3 complex, to a phenylalanine using PyMOL (Molecular Graphics System, Version 1.5.0.1 Schrödinger, LLC). The initial coordinates of the VDR-MK complex were generated by mutating the VDR_H305F-MK F305 residue to H305.

The initial structure used to simulate the MD of the helix 12 closed, apoVDR was generated by removing 1,25D3 from the X-ray coordinates of VDR-1,25D3 complex (pdb: 1DB1). The initial structure of helix 12 closed apoVDR (H305F/H397F) was constructed by mutating both H305 and H397 of pdb: 1DB1 (Additional file 1:Table S1).

The initial structure of the helix 12 opened, apoVDR molecule was generated by homology modeling, in which the last twelve residues of the minimized VDR (aa414-427) were aligned to the last twelve residues of the apoRXR (PDB: 3OZJ). It is noted that an attempt to align aa401-427 failed using this method; however, the apoVDR model generated does allow for the assessment of the effect helix 12 being positioned away from the core of the VDR ligand binding domain has on the MD simulations. All models addressed in this study are listed in Additional file 1: Table S1.

### Molecular models and computational details

We performed molecular dynamics (MD) simulations using the Amber11 and NAMD2.6 simulation packages with the ff99sb amber force field for the proteins (Okur et al 2003; Hornak et al 2006; Phillips et al 2005; Cornell et al 1995). The parameters for the ligands were generated with the Antechamber module of the AMBER11 package using the general amber force field (gaff) (Wang et al 2006). The root mean square deviation (RMSD) for all models is shown in Additional file 2: Figure S1. Before solvating the system, the hydrogen atoms, side chains and the protein were each minimized for 1000 steps using a combination of steepest descent and conjugate gradient. Sodium counter ions were added to the minimized protein and the system was solvated in a cubic shell using the TIP3PBOX water model. The size of the water box for VDR protein was 82x79x89 Å^3^. The water box was minimized for 1000 steps while the protein was held fixed. Subsequently, the water box was equilibrated at 200K for 10 ps. The complex was gradually warmed up from 200K for 2500 steps followed by 5 ps of simulation at 250K.

### The molecular dynamics simulations for the following models were performed at 300K

32ns for VDR-LBD-1,25D3 complex, 17ns for the three mutated analogues, 28 ns for the homology modeled apoVDR, 25 ns for apo VDR helix 12 closed, 15ns for apoVDR_FF helix 12 open, 21 ns for apoVDR_FF helix 12 closed, and 23ns for the VDR-MK complex (Additional file 1: Table S1). The 32 ns molecular dynamic stimulation for 1,25D3 bound to VDR was performed with two different starting models: (a) the X-ray conformation of the protein with crystal water molecules 502–511 included and (b) the X-ray conformation of the protein without the crystal water molecules*.* The phi entropy and the side-chain entropy values were computed using the Gibbs equation and the T-Analyst program (Ai et al 2010; Chang et al 2008).

The pKa of the histidine residues in the protein were calculated *by* Multi-Conformation Continuum Electrostatics (MCCE) (Georgescu et al 2002; Song et al 2009). The results predict that both histidine residues near the binding site, HIS305 and HIS397, are only protonated at a pH below 6. The only histidine side-chains that are protonated at physiological pH levels are HIS140 of helix 1 and HIS326 and HIS330 of helix 7. All three of these histidine groups are exposed to solvent and reside close to an acidic side-chain. Since these histidine residues are far from the binding site their protonation state was not altered in our MD simulations.

The energy of interaction between the ligand and the protein was calculated by the Molecular Mechanics/Generalized Born Surface Area (MM/GBSA) method (Tsui and Case 2000; Bashford and Case 2000; Hou et al 2011) using an in-house script with the GBSA module of the AMBER10 package.

## Results

### Effect of VDR mutations and MK side-chain chemistry on VDR charge clamp distance

Transactivation by a holo-NR requires the formation of a charge clamp that grips the LXXLL helix of nuclear coactivators. This interaction occurs on the surface of the NR ligand binding domain (LBD) (Glass and Rosenfeld 2000). In CV1 cells co-transfected with VDR and VDRE-SEAP reporter constructs, 1,25D3 was measured to have a similar EC_50_ in the H305F and H305F/H397F (VDR_FF)-1,25D3 complexes when compared to hVDRwt-1,25D3. In VDR_H397F, 1,25D3 was a 10-fold weaker agonist ligand. Conversely, the hVDR-1,25D3 antagonist, MK, functions as a genomic superagonist in VDR_H305F and VDR_FF transfected cells (Mizwicki et al 2009a).

**Figure 2:**
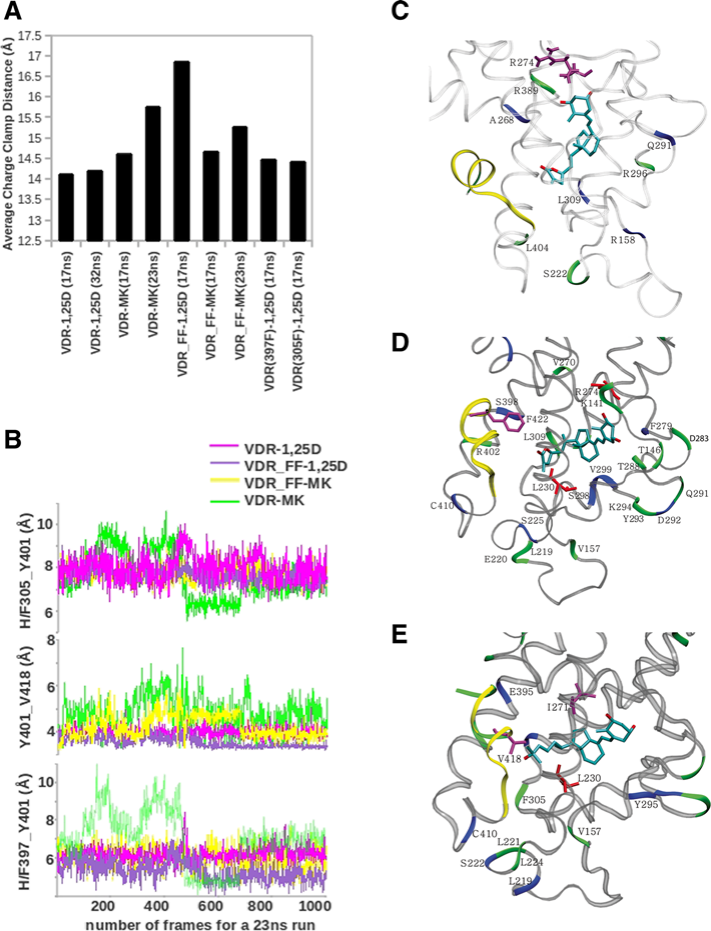
**Molecular dynamics (MD) simulations of holo VDR complexes. A)** The average charge clamp distance between K246 and E420 is shown for the holoVDR models (also see supplemental Fig. 3C). **B)** The distance in relation to time, between H397 and Y401 (bottom panel), Y401 and V418 (middle panel) and H305 and Y401 (top panel) during the 23ns MD run is plotted for VDR-1,25D3 (magenta), VDR_FF-1,25D3 (purple), VDR-MK (light green) and VDR_FF-MK (yellow). The backbone and ligand binding pocket (LBP) side-chain dynamics of **C)** VDR-MK compared to VDR-1,25D3 (Table 1), **D)** VDR_FF-MK compared to VDR-MK and **E)** VDR_FF-1,25D3 compared to VDR-1,25D3. Regions of the VDR where the backbone flexibility is increased or decreased are indicated by coloring the ribbon light green and blue respectively. Changes in backbone flexibility are only mentioned for the residues with the entropy difference > 0.14 kcal/mol. In these panels more flexible or rigid side-chains are rendered in tube structure, colored magenta and red respectively. The flexibility changes for side-chains within the LBP are only mentioned when the entropy difference was above 0.3 kcal/mol. In these panels H12 is colored yellow and only the lower portion of the VDR is shown for clarity (compare to Figure 1).

VDR-1,25D3 molecular dynamics (MD) simulations demonstrated that the average charge clamp distance between the side chain nitrogen of K246 and the delta carbon of E420 remained constant over a 32ns run (Figure 2A and Additional file 3: Table S2a). Similar average charge clamp distances were observed in the VDR_H305F-1,25D3 and VDR_H397F-1,25D3 models. Consistent with the functional results, the VDR_FF-MK charge clamp distance was observed to be more similar to the hVDRwt-1,25D3 than the VDRwt-MK model. In the latter model, the charge clamp equilibrated to be over 1.5 Å greater than VDRwt-1,25D3 (Figure 2A). The one model that did not correlate with functional data was the VDR_FF-1,25D3 model, where the charge clamp distance was on average 2.5 Å greater than VDR-1,25D3 (Figures 1 and 2A).

It is noted that measuring the charge clamp distance does not directly correlate with NCoA affinity; however, it does provide a read-out for how the bimolecular interaction between the VDR and its’ ligand can alter the MD of surface regions linked to macromolecular complex formation (Vaisanen et al 2002). It is also noted that recent mass spectrometry coupled hydrogen-deuterium exchange results showed that when VDR binds DNA, it alters the backbone flexibility of the VDR nuclear coactivator surface in the intact VDR-RXR complex (Zhang et al 2011).

### Changes in helix 11 and 12 intramolecular interactions in the holoVDR models

The closed helix 12 conformation is in part stabilized by a T-shaped pi-pi interaction made between H397 and F422 and a displaced parallel pi-pi interaction between H397 and Y401 (Additional file 4: Figure S2). These intramolecular interactions aid in stabilizing the closed conformation of helix 12 and are enhanced by the terminal end of the 1,25D3 side-chain forming an H-bond with H397. When the distance between H397 and Y401 in all the models was monitored, only the VDR-MK model showed significant fluctuations (Figure 2B, bottom panel). More dynamic fluctuations between V418 and Y401, and H305 and Y401 in the VDR-MK model were also observed (Figure 2B, middle and top panels). Lastly, the distance between H397 and V418 was on average 1.1 Å larger in the VDR-MK model when compared to the other models. This is important because H397 is a helix 11 residue and V418 a helix 12 residue; therefore, increase in this distance indicates that helix 12 may be moving away from helix 11. Coupled the distance calculations confirm that the residues that exclusively contact the ligands side-chain atoms in the VDR LBP (e.g. C-terminus of helix 11 and helix 12) become more flexible when complexed to MK (Perakyla et al [2004]).

In general the fluctuations in the VDR_FF models were lower in all cases when compared to the VDRwt models (Figure 2B). This is due to the enhanced pi-pi intramolecular interaction energy between F305 and Y401 and F397 and F422. F305 is towards the back of the VDR LBP (Figure 1), near helix 7, H397 and Y401 are two residues towards the C-terminus of helix 11 and F422 is part of helix 12. Thus optimizing pi-pi interactions between these residues provides an allosteric link between the regions of the VDR that bind RXR (the back), ligand (the middle) and helix 12, the front of the VDR (Figure 1).

### Overall backbone and side-chain entropy changes in the holoVDR models

The dihedral entropy calculation is one way to directly measure the flexibility of protein backbone and side-chains (Zhang and Liu 2006). The backbone entropy over the 23 ns VDR-MK MD increased for R296, L404 and G423, while L309 became more rigid when compared to VDR-1,25D3 (Figure 2C). Of these residues L309 and L404 have been shown by a number of laboratories to provide important hydrophobic, vdW contacts required for VDR-1,25D3 activation of gene transcription (Mizwicki et al 2009a; Choi et al 2003; Yamamoto et al 2006).

When both H305 and H397 of VDR-MK were mutated to phenylalanine (VDR_FF), the backbone atoms of helix 7 (V297 and S298), helix 11 (S398) and helix 12 (C410) became more rigid when compared to VDRwt-MK (Figure 2D). Alternatively, the backbone atoms of helix 1 (K141), helix 3n (L219 and E220), the β-sheet (K294), helix 5 (V270), helix 7 (L309 and E311) and helix 11 (R402) became more flexible (Figure 2D). The increased flexibility of R402 was not expected given, previous proteolytic digest experiments indicated that trypsin cleavage at R402 was significantly attenuated by the VDR_FF double mutation (Mizwicki et al 2007). The backbone motions revealed by the VDR_FF-1,25D3 MD simulation indicated that the β-sheet region, C-terminal end of helix 11 (Q400), helix 12 (L419 and F422) and F305 became more flexible. Alternatively, helix 7 (L309), helix 10 (E395) and helix 12 (C410) residues became more rigid (Figure 2E). Thus a common feature of the FF mutation was to increase the rigidity of C410 and residues juxtaposed to H397.

Comparison of the 1,25D3 and MK VDRwt LBP side-chain movements demonstrated that only the side-chain of R274 (helix 5 C-terminus) was significantly more flexible when bound to MK (Figure 2C). The VDR_FF mutation caused the side-chain of F422 (helix 12) to become more flexible and L230 (helix 3) and R274 (helix 5) to become more rigid when complexed to MK (Figure 2D). Thus the major difference in the VDR side-chain entropy during the VDRwt-MK and VDR_FF-MK simulations was observed in the A-ring region of the VDR LBP, not the helix 11 and helix 12 regions of the LBP. Lastly, the VDR_FF-1,25D3 side-chain entropy results demonstrated that the FF mutation caused I271 and V418 to become more flexible and L230 to become more rigid (Figure 2E). All of the residues highlighted above were shown by VDR ALA scanning analysis to be crucial to VDR agonist function (Yamamoto et al 2006).

Collectively, the MD results for VDR-MK suggest that MK functions as an effective antagonist of hVDRwt because it does not stabilize the R274 side-chain and MK allows the C-terminal backbone atoms of helix 11 and helix 12 become for flexible. Thus the helix 11 and helix 12 MD results are consistent with previous flexible docking and limited proteolytic digest studies that showed increased disorder in this region is produced when MK is bound to hVDRwt (Mizwicki et al 2009a). Furthermore, recent VDR-MK hydrogen-deuterium exchange coupled mass spectroscopy results demonstrated that the amide backbone exchange for peptides mapping to helix 3, residues 286–316 and 384–419 showed significantly enhanced exchange in the VDR-MK complex (Mizwicki et al 2012).

### Comparison of 1,25D3 and MK MD and Interactions with VDR LBP Residues

**Figure 3:**
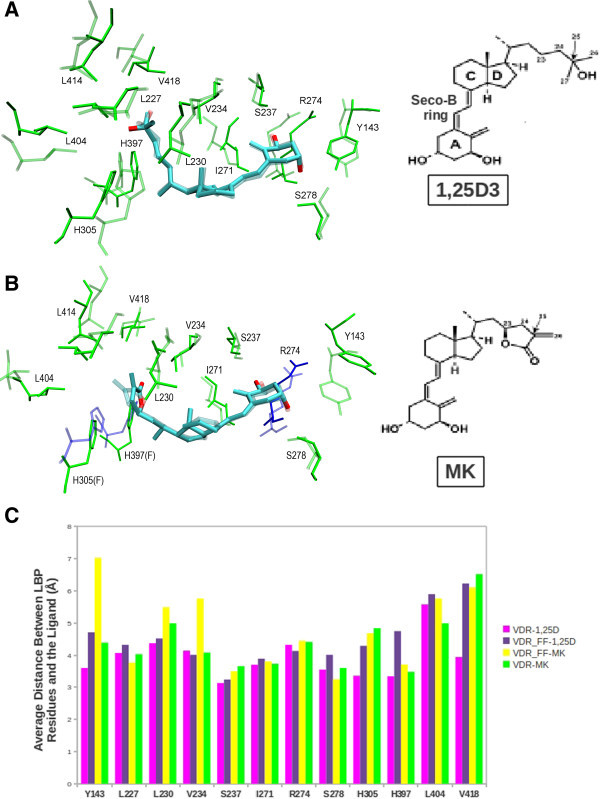
**Molecular dynamics of ligand binding pocket. A)** Comparison, superimposition, of the VDR-1,25D3 and VDR_FF-1,25D3 complexes. In the figure, the VDR-1,25D3 model is shown as solid, green bonds with R274, H305 and H397 (blue). The VDR_FF-1,25D3 model is shown as transparent, green and blue (only R274) bonds. The chemical structure of 1,25D3 is shown to the right of the model and the A-ring, seco-B-ring, C/D-ring and side-chain 25-OH group are labeled. **B)** Superimposition of the VDR-MK and VDR_FF-MK molecular models. The binding site of VDR-MK is shown as transparent, green bonds and the binding site for VDR_FF-MK is shown as solid bonds. As in panel **A**, R274, H305 and H397 are colored blue and F305 and F397 green. The chemical structure of MK is shown to the right of the figure and carbon-25 labeled. **C)** The graph shows the mean, averaged hydrogen bonding and hydrophobic distances between the binding site residues and the ligand for VDR-1,25D3 (magenta), VDR-MK (green), VDR_FF-1.25D3 (purple) and VDR_FF-MK (yellow).

Common structural features of MK and 1,25D3 are their A-ring, seco-B and CD-ring chemistries. They differ in the structures of their side-chains. 1,25D3 terminates with a hydroxyl group attached to two methyl groups, while the side-chain of MK terminates with an exo-cyclic, α,β-unsaturated lactone (Figure 3A). As could be implied from the relatively few VDR-MK backbone and side-chain entropy changes (Figure 2C), the A-ring (β-chair), seco-B-ring (6-*s*-*trans*) and CD-ring (transhydrindane) geometries of 1,25D3/MK remained similar during the VDR-1,25D3/MK MD simulations. In addition, both the 1,25D3 and MK side-chains formed a hydrogen bond with H397, but only 1,25D3 formed a hydrogen bond with H305 (Figure 3A and B). In the VDR-MK complex, the lactone ring of MK remained parallel to H305 (Figure 3B) during the MD run.

In an attempt to further our understanding for how the VDR_FF mutation converts MK, but not 1,25D3 into a superagonist, the average nearest neighbor hydrophobic and hydrogen bonding distances between residues composing the LBP of four models were compared (Figure 3C and Additional file 3: Table 2b and c). In the VDR_FF-MK complex only Y143 and V234 were on average 2.64 Å and 1.68 Å further away from MK when compared to VDRwt-1,25D3. Conversely, over the 23 ns VDR_FF-1,25D3 MD run, the Y143, V418, 305 and 397 residues were on average 1.11 Å, 2.29 Å, 1.48 Å and 1.41 Å further away from 1,25D3 than in VDRwt. Furthermore, comparison of the VDR_FF-1,25D3 and MK simulations showed that MK remained on average 1.04 Å closer to F397. This interaction may contribute to the superagonist effect of MK, because it is known that the intramolecular interaction between the H397 side-chain and F422 (helix 12) is important VDR activation of gene transcription (Gonzalez et al 2003).

### Interaction energy between 1,25D3 or MK and VDRwt, VDR_H305F or VDR_FF

The average interaction energies obtained from MMGBSA calculations are provided in Table 1 for all the holoVDR complexes. The results demonstrated that the van der Waals energy of interaction dominated over the electrostatic energy of interaction in all complexes, highlighting the weighted importance of the hydrophobic residues composing the LBP in providing binding strength. The 1,25D3 electrostatic interaction is more stable in VDRwt than the H305 and H397 mutants (Table 1). MK established a stronger electrostatic energy of interaction than 1,25D3 with all of the VDR molecules (Table 1), even though it formed only one H-bond with H397 (Figure 3B). The MK-H397 H-bond was also observed in the MK-H305F x-ray structure (Kakuda et al 2010). The polarization interaction component of the Interaction Energy (▵E_pol_, Table 1) was most repulsive in VDR-MK. Weaker ▵E_pol_’s were observed for the VDR_FF-MK and VDR-1,25D3 MD runs (Table 1). Thus these results indicate that energetically, MK’s switch from an antagonist into an agonist VDR ligand is nested in a high ▵E_pol_ value in hVDRwt being reduced by the FF mutation.

**Table 1:** **Interaction energies from MMGB/SA calculations results for the holo VDR models**

	▵Evdw	▵Eeel	▵Epol	▵Esas	Total(kcal/mol)	Binding Affinity**(nM)
VDR-1,25D3	-65.24+/-5.10	-10.43+/-2.31	5.15+/-5.15	-4.23+/-0.12	-74.76+-3.01	5.21+/-2.79
VDR(H305F)-1,25D3	-66.52+/-2.62	-11.03+/-2.65	6.44+/-1.10	-4.27+/-0.10	-75.38+/-2.93	17.0+/-2.74
VDR_FF-1,25D3	-66.29+/2.63	-7.95+/-2.33	5.93+/-1.25	-4.25+/-0.12	-72.55+/-2.72	85.4+/-36.9
VDR-MK	-64.83+/-2.65	-14.39+/-2.73	8.55+/-1.53	-4.43+/-0.15	-75.12+/-3.12	37.9+/-8.14
VDR_FF-MK	-59.264+/-2.44	-13.86+/-2.35	4.60+/-1.16	-4.19+/-0.12	-72.72+/-2.84	73.5+/-12.0

ΔE_vdw_ is the van der waals interaction energy. ▵E_eel_ is the electrostatic interaction energy. ▵E_pol_ is the polar solvation free energy. ▵E_sas_ is the solvent accesible surface area energy.

### Effect of water on ligand binding pocket MD

X-ray results all show a ‘kinked’ conformation of R274 that is stabilized by formation of an H-bonds with the 1α-OH group and crystallographic water (Hourai et al 2006), suggestive that water plays a key role in aiding in the stability of the local geometry of the active R274 side-chain conformation. The VDR MD simulations outlined above were all performed in explicit solvent and were shown to correlate quite well with 1,25D3 and MK functional results; therefore, we next used the MD technique to further investigate the relative importance of the x-ray water molecules interacting with R274. This was accomplished by running 32 ns MD simulations on two different VDR-1,25D3 starting models: (**a**) the X-ray conformation of the protein with crystal water molecules 502–511 included and (**b**) the X-ray conformation of the protein without the crystal water molecules (i.e. the technique used exclusively to this point).

**Figure 4:**
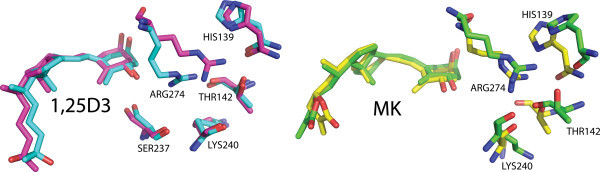
**The role of side-chain chemistry on ARG274 conformational flexibility.** **A)** The conformation of ARG274 and its hydrogen bonding partners for VDR-1,25D3 at 1.0 ns and 30.0 ns are shown in magenta and light blue respectively. **B)** The conformation of ARG274 and its hydrogen bonding partners for VDR-MK at 1.0 ns and 23.0 ns are shown in light green and yellow respectively. Water molecules are not shown for clarity.

In both of the models, the side-chain hydroxyl group of 1,25D3 hydrogen bonds to H305 and H397; the 3β-hydroxy of 1,25D3 is on average 3.5 Å away from the side chain hydroxyl group of S278 and Y143; and the 1α-hydroxy is on average 3.1 Å away from S237. In model **a**, R274 hydrogen bonds to the 1α-hydroxy of 1,25D3 and three crystal water molecules and over the duration of the MD run did not move from the kinked conformation. In model **b**, two water molecules were observed between the guanidine head group of R274 and the 1α-hydroxy of 1,25D3 to begin the simulation. One (ns) into the simulation, the carbon chain of R274 moved into a staggered, extended geometry (see Additional file 5: Figure S3) and the 3β-hydroxy of 1,25D3 was proximal to the backbone carbonyl oxygen of R274 (Figure 4A). Between 10 and 20 ns, the two water molecules became displaced from the R274 residue, but the side-chain remained staggered and in close hydrogen bonding distance to H139 and T142 (Figure 4A). These two residues are not actually part of the ligand binding domain, but rather they belong to the hinge domain of the VDR molecule. Thirty (ns) into the MD run the R274 R-group lost contact with H139, and rotated into the ‘kinked’ geometry where the guanidine head group formed the hydrogen bond with the 1-OH group of 1,25D3 observed in the x-ray structures (Figure 4A). During the VDR-MK MD run, it was observed that the extended geometry of R274 became ‘kinked’ 8 ns into the simulation; however, the guanidine head group did not get within H-bonding distance to the 1α-OH group of MK (Figure 4B and Additional file 6: Figure S4). Instead the R274 polar side-chain remained in a hydrogen bond network with H139, K240 and/or T142 (Figure 4B).

### The effect of helix 12 conformation on apoVDR molecular dynamics

**Figure 5:**
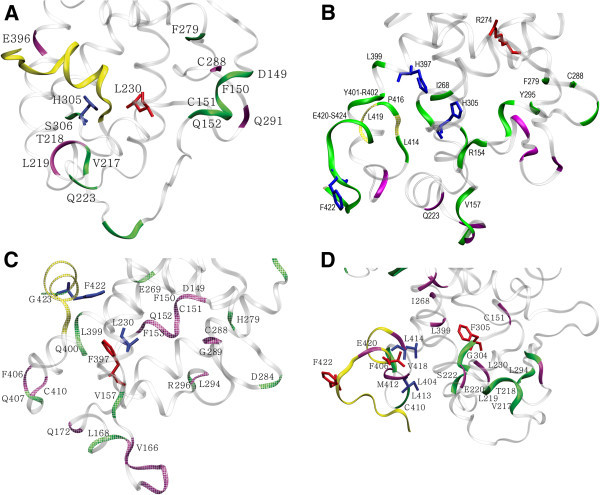
**Changes in the backbone and side-chain molecular dynamics (MD) of apoVDR complexes.** In the figure panels helix 12 is colored yellow; increased (green ribbon) or reduced (magenta ribbon) backbone flexibility; and increased (red tube structure) or reduced (blue tube structure) side-chain flexibility are highlighted and labeled. **A)** Changes in the MD when helix 12 is closed and the 1,25D3 ligand removed (apoVDR) when compared to VDR-1,25D3. **B)** Changes in the flexibility of the homology modeled helix 12 opened apoVDR moleclule backbone and side-chain residues when compared to the helix 12 closed apoVDR molecule. **C)** Changes in the flexibility of the backbone and side-chain atoms of the closed helix 12 apoVDR_FF conformation when compared to the helix 12 closed, apoVDR conformer. **D)** Changes in the backbone and side-chain flexibility of the helix 12 opened, apoVDR_FF conformation when compared to helix 12 opened apoVDR conformer. As in Figure 2, only changes in the backbone flexibility of residues with an entropy difference > 0.14 kcal/mol are labeled in the figure. Flexibility changes of LBP side-chain residues are labeled if the entropy difference was > 0.3 kcal/mol in the comparsion. Only the lower portion of the VDR is shown for simplicity (compare to Figure 1).

Removal of 1,25D3 from VDRwt resulted in no major overall change in the ligand binding pocket (LBP) backbone entropy. Perhaps most importantly, helix 12 did not show a significant change in backbone motion in the absence of ligand when compared to VDR-1,25D3 (Figure 5A). Overall, not many changes in the VDR side-chain flexibility was observed; however, H305 did become more flexible and L230 more rigid during the run (Figure 5A).

As expected, when helix 12 was homology modeled in the opened conformation (see methods), the backbone entropy calculations showed many more changes in flexibility over the run. For example, the backbone atoms of L230, R274, F279 and the C-terminal end of helix 11 (L399, Y401 and R402) became significantly more flexible (Figure 5B). According to the side-chain entropy calculations, opening of helix 12 also increased the flexibility of L230, H397 and F422 and reduced the flexibility of R274 (Figure 5B). These results suggest that removal of the LBP lid (i.e. helix 12) increases the backbone flexibility of helicies 3, 5 and 11, but increases the rigidity of the R274 side-chain. The latter result can be potentially explained by the evidence presented herein that in the absence of ligand, R274 would bias the local conformation where the R274 side-chain interacts with H139 and T142.

Mutating H305 and H397 (apoVDR_FF) caused the helix 11 and 12 backbone residues (i.e. L399, L414, M412, K413 and E420) to become more rigid when helix 12 was opened (compare Figure 5C and D). Alternatively the backbone atoms of F406 and C410 became more rigid, and L399, Q400 and Q407 became more flexible when helix 12 was closed (Figure 5C). The side-chain entropy calculations indicated that L230 and F422 side-chains became more flexible and H397 more rigid, when helix 12 was closed (Figure 5C). Whereas, the side-chain flexibility of L404 and L414 and made V418 and F422 become more rigid (Figure 5D). Thus the results suggest that the VDR_FF mutant had a greater stabilizing effect on the opened helix 12 conformation rather than the closed conformation.

## Discussion

Much is known about the thermodynamically favored conformation of the nuclear vitamin D receptor (VDR) and its’ role as a nuclear transcription factor; however, little is understood about its’ intrinsic flexibility (Mizwicki et al 2007) and how VDR molecular ensembles may influence a given ligands affinity and function. Herein, molecular dynamics simulations were used to assess a) the molecular events dictating whether MK functions as a VDR antagonist or superagonist ligand; b) whether x-ray water is required to accurately simulate the bimolecular interaction between the VDR and ligand; and c) the effect removal of ligand, the opening helix 12 and mutation of H305 and H397 have on the VDR backbone and side-chain flexibility.

Evidence that the VDR-MK MD results, performed using an explicit solvation model, correlate well with MK structure-function results included a) the side-chain of R274 is the most flexible residue in the LBP of VDR when bound to MK; b) the backbone of K246, one of the charge clamp residues (Figure 1), becomes significantly more flexible and the clamp opens (Figure 2A); and c) the backbone entropy of helix 11 and helix 12 increases (Figure 2C). Consistent with previous flexible docking results (Mizwicki et al 2009a), the MD runs also show that the side-chain of MK preferentially moves closer to L227 (helix 3) and L404 (helix 11), and away from helix 12, i.e. L414 and V418 (Figure 3C). The MD results also show that the migration of MK away from helix 12 and towards the C-terminal end of helix 11 is facilitated by the pi-pi interaction made between the MK lactone ring and H305. Migration of the MK lactone side-chain places the exo-cyclic methylene as close as it could possibly get to C403 in the x-ray VDR conformation. This movement of the MK side-chain towards L404 could increase the probability for the formation of a Michael adduct C403 (Mizwicki et al 2012; Kakuda et al 2010).

The conversion of MK into a VDR superagonist in VDR_FF has been postulated to be triggered by the intermolecular interaction between the F305 and the lactone ring of MK (Mizwicki et al 2012; Kakuda et al 2010). In support of this hypothesis, the VDR_FF-MK and 1,25D3 MD results show they have similar average charge clamp distances (Figure 2A), intramolecular interactions between helix 11 (H397 and Y401) and helix 12 (V418 and F422) side-chains (Figure 2B) and backbone/side-chain entropy. Importantly, the MD simulations demonstrate that F397 is significantly further away from 1,25D3 when compared to VDRwt, but when bound to MK, F397 and F422 become closer to one another. Overall analysis of the MD frames indicates that movement of F397 (helix 11) away from 1,25D3 is caused by F397 gravitating toward F422 (helix 12). This motion provides an explanation for why VDR_FF-1,25D3 has an increased charge clamp distance and why the electrostatic interaction between 1,25D3 and the VDR_FF complex is significantly reduced, whereas it is not for MK (Table 1).

A novel finding from the MM/GBSA interaction energies was that no real change in total interaction energy is observed for 1,25D3 or MK throughout the various MD runs; however, dramatic changes in the relative contributions of each term to the total interaction energy are observed. For example, MK had a weaker hydrophobic interaction (▵E_vdw_), stronger electrostatic interaction (▵E_eel_) and greater polar solvation free energy (▵E_pol_) with VDRwt, when compared to 1,25D3 (Table 1). This indicates that the reduced vdW interaction likely drives the reduced affinity of MK for the VDR in a steroid competition assay (Mizwicki et al 2009a). Combining this physical characteristic with an increased ▵E_pol_ may be a signature that can be used in the design of future VDR antagonists (Table 1).

While we were unable to homology model the apoRXR-like opened LBD conformation, peeling of helix 12 away from the body of the LBD enhances the side-chain entropy of H305 and fractures the displaced parallel pi-pi interaction between H397 and Y401. In the closed helix 12 VDR MD run, H397 maintains the intramolecular interaction with Y401, perhaps explaining why the H397 side-chain entropy is similar in the helix 12 closed *apo* and *holo* models. It is noted that a recent variant of VDR_FF, where H397 was mutated to a Tyr rather than a Phe, showed enhanced sensitivity for vitamin D_3_, which has no activity in VDRwt (Ousley et al 2011).

Opening of helix 12 also causes the residues of helix 11 and 12 to become significantly more flexible and gravitate toward the LBP; however unlike the C-terminal portion of helix 11 in the apoRXR crystal structure (Bourguet et al 1995), H11 residues did not enter the region of the LBP normally occupied by the seco-B and A-ring of the ligand. It has been proposed that the ability of the FF mutation to greatly protect the apoVDR against trypsin cleavage by increasing the rigidity of the closed helix 12 conformation by stabilizing residues proximal to the two C-terminal trypsin sites, R402 (helix 11) and K413 (helix 12) (Mizwicki et al 2007). However the MD results demonstrate that the backbone entropy of residues proximal to R402 and K413 was reduced when helix 12 was opened, rather than the apo-helix 12 closed conformer (Figure 5C and D). This result suggests that the FF mutations ability to protect against helix 12 libation may be through its’ altering the stability of other VDR conformational ensemble members than the helix 12 closed conformer. The fact that both the apo-helix 12 opened and closed VDR and VDR_FF MD runs show oscillation about a an average RMSD (Additional file 2: Figure S1) support the theory that the closed helix 12 conformation of VDR is sampled in the absence of 1,25D3 and therefore a conformational ensemble model should be applied in rationalization/translation of VDR structure-function results (Mizwicki and Norman 2009b).

Perhaps the most intriguing and novel finding in this study was that the replacement of x-ray water molecules with explicit water did not alter the accuracy in simulating the MD that exist between VDR and 1,25D3/MK.; even though, removal of the x-ray water allows the R274 side-chain to sample multiple conformational states in the presence of ligand. Given the kinked, x-ray geometry of the R274 side-chain is induced by 1,25D3 during the VDR-1,25D3 MD run, it is plausible that shifting the equilibrium of the R274 side-chain to favor the kinked geometry is key to the molecular switch underpinning the activation of VDR transcription. Screening of additional ligands and extending simulation times will allow for future testing of this hypothesis. In closing our MD, dihedral entropy and interaction energy calculations confirm and build upon previous structure-function studies and provide novel findings whose true value will be defined as we generate a greater understanding of the molecular dynamics underpinning VDR ligand recognition and dissociation and the apoVDR ensemble model.

## Electronic supplementary material

Additional file 1: **Table S1.** The model systems used in this study. Brief description, the system name, initial PDB structure code, total simulation time and number of atoms for the ten apo- or holo VDR models are summarized in the table. (*) indicates the pdb file was modified. The system names listed here are use throughout the figures and text. (JPEG 359 KB)

Additional file 2: **Figure S1.** RMSD plots. (A) for the binding site residues (I127, L230, V234, S237, I272, R274, S278, H305, H397 and V418) VDR-1,25D (pink), VDR_FF-1,25D (purple), VDR-MK (green) and VDR_FF-MK (yellow). (B) for residues 130-400, VDR-1,25D (pink), VDR_FF-1,25D(light purple), VDR-MK (green) and VDR_FF-MK (yellow). (C) for residues 130-400, closed apo VDR (blue), h12 open apo VDR (light blue), h12 closed apo_FF (gray) and h12 open apo_FF (light red). (JPEG 126 KB)

Additional file 3: **Table S2.** The distance (A) between the binding site residues of VDR and 1,25D/MK and the distance between the charge clamp residues. (a) the distance between the charge damp residues in all the models. (b) the distance between the binding site residues and the ligand that are involved in hydrogen bonding. (c) the distance between the binding site residues and the ligand that are involved in hydrophobic interaction. (JPEG 185 KB)

Additional file 4: **Figure S2.** Helix 11 and helix 12 flexibility in the holo VDR models. HIS/PHE397, TYR401, VAL418 and PHE422 are shown for the VDR-1,25D, VDR_FF-1,25D, VDR_MK and VDR_FF-MK model in magenta, purple, light gren and yellow. (JPEG 189 KB)

Additional file 5: **Figure S3.** Rotameric states for the side-chains of ARG274 in VDR-1,25D. (a) 17ns run, (b) 32ns run, (c) 32ns run with crystal waters included. SC1, SC2, SC3 and SC4 are shown in magenta, yellow, green and blue respectively. (JPEG 347 KB)

Additional file 6: **Figure S4.** Rotameric state for side-chains of ARG274 in VDR-MK. (a) 17ns run, (b) 23ns run. SC1, SC2, SC3 and SC4 are shown in magenta, yellow, green, and blue respectively. The second side-chain of R274 in model a has a 10% occupancy at 180° and 2% occupancy at 90°, while in model **b** there is a 20% occupancy at 90°. (JPEG 357 KB)
