# Supplementary figures and images for: Agonist and antagonist binding to the nuclear vitamin D receptor: dynamics, mutation effects and functional implications
Source: In Silico Pharmacol. 2013 Feb 12;1:2. doi: 10.1186/2193-9616-1-2 (PMC4215818; doi:10.1186/2193-9616-1-2)

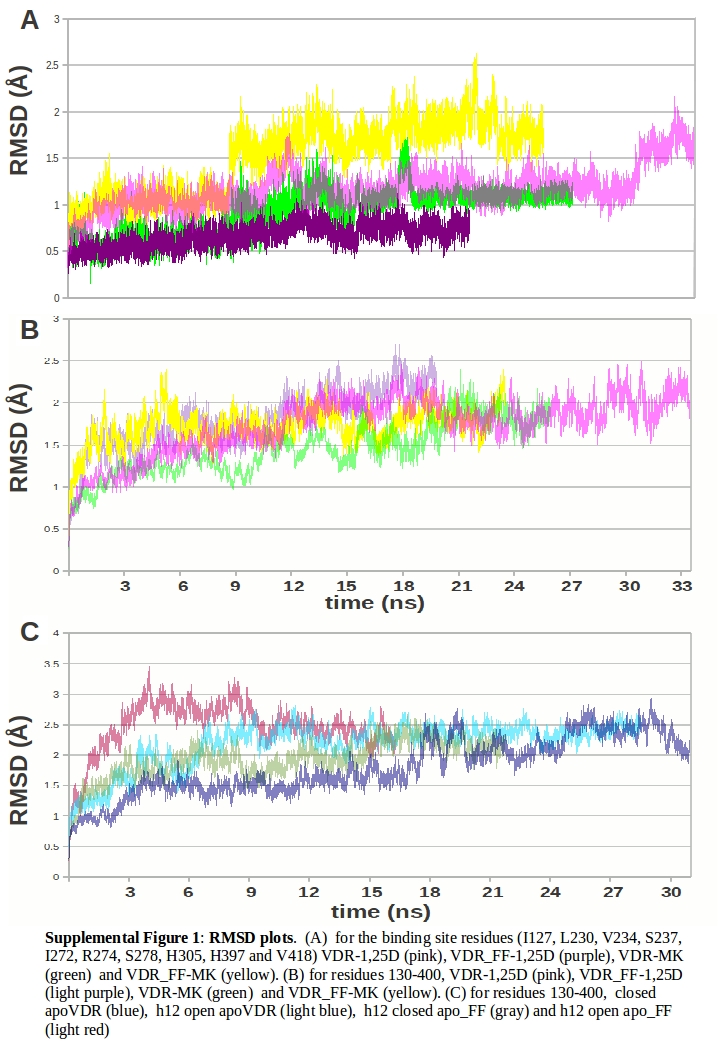

Supplement: Supplementary file 1 — Additional file 1: Table S1. The model systems used in this study. Brief description, the system name, initial PDB structure code, total simulation time and number of atoms for the ten apo- or holo VDR models are summarized in the table. (*) indicates the pdb file was modified. The system names listed here are use throughout the figures and text. (JPEG 359 KB) [file 40203_2012_2_MOESM1_ESM.jpeg]

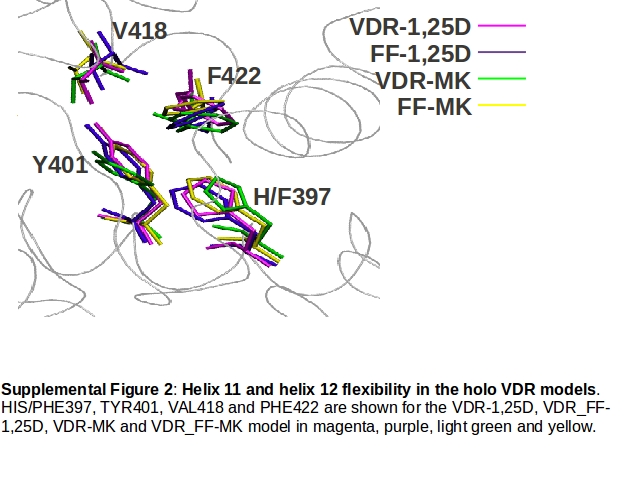

Supplement: Supplementary file 2 — Additional file 2: Figure S1. RMSD plots. (A) for the binding site residues (I127, L230, V234, S237, I272, R274, S278, H305, H397 and V418) VDR-1,25D (pink), VDR_FF-1,25D (purple), VDR-MK (green) and VDR_FF-MK (yellow). (B) for residues 130-400, VDR-1,25D (pink), VDR_FF-1,25D(light purple), VDR-MK (green) and VDR_FF-MK (yellow). (C) for residues 130-400, closed apo VDR (blue), h12 open apo VDR (light blue), h12 closed apo_FF (gray) and h12 open apo_FF (light red). (JPEG 126 KB) [file 40203_2012_2_MOESM2_ESM.jpeg]

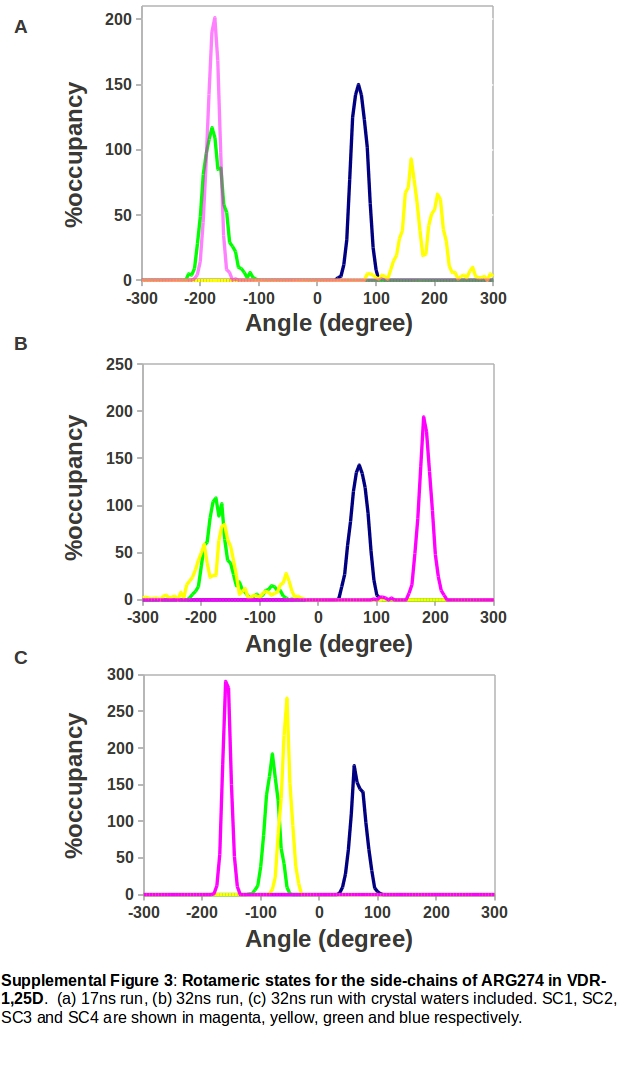

Supplement: Supplementary file 3 — Additional file 3: Table S2. The distance (A) between the binding site residues of VDR and 1,25D/MK and the distance between the charge clamp residues. (a) the distance between the charge damp residues in all the models. (b) the distance between the binding site residues and the ligand that are involved in hydrogen bonding. (c) the distance between the binding site residues and the ligand that are involved in hydrophobic interaction. (JPEG 185 KB) [file 40203_2012_2_MOESM3_ESM.jpeg]

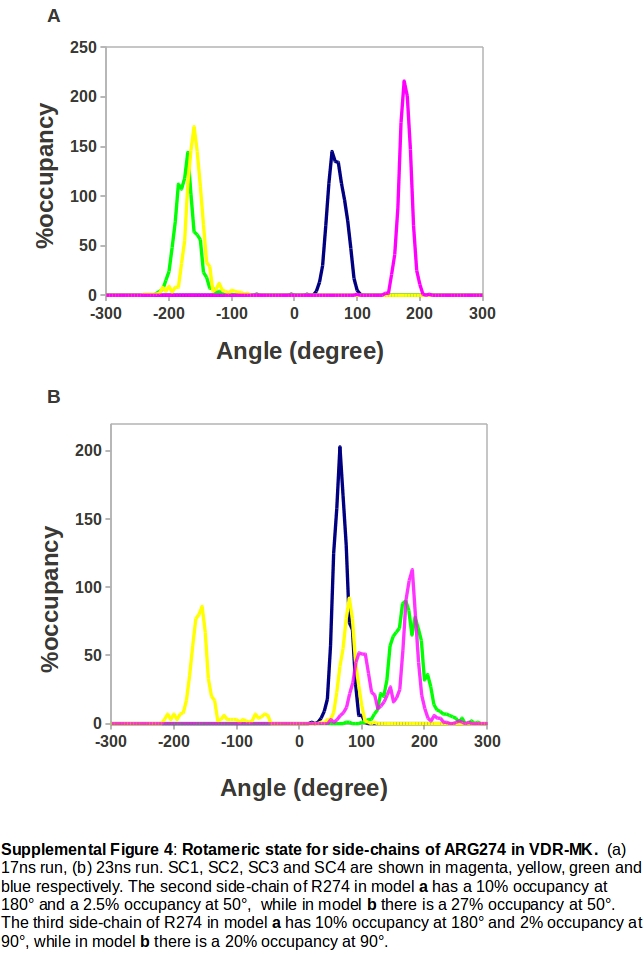

Supplement: Supplementary file 4 — Additional file 4: Figure S2. Helix 11 and helix 12 flexibility in the holo VDR models. HIS/PHE397, TYR401, VAL418 and PHE422 are shown for the VDR-1,25D, VDR_FF-1,25D, VDR_MK and VDR_FF-MK model in magenta, purple, light gren and yellow. (JPEG 189 KB) [file 40203_2012_2_MOESM4_ESM.jpeg]

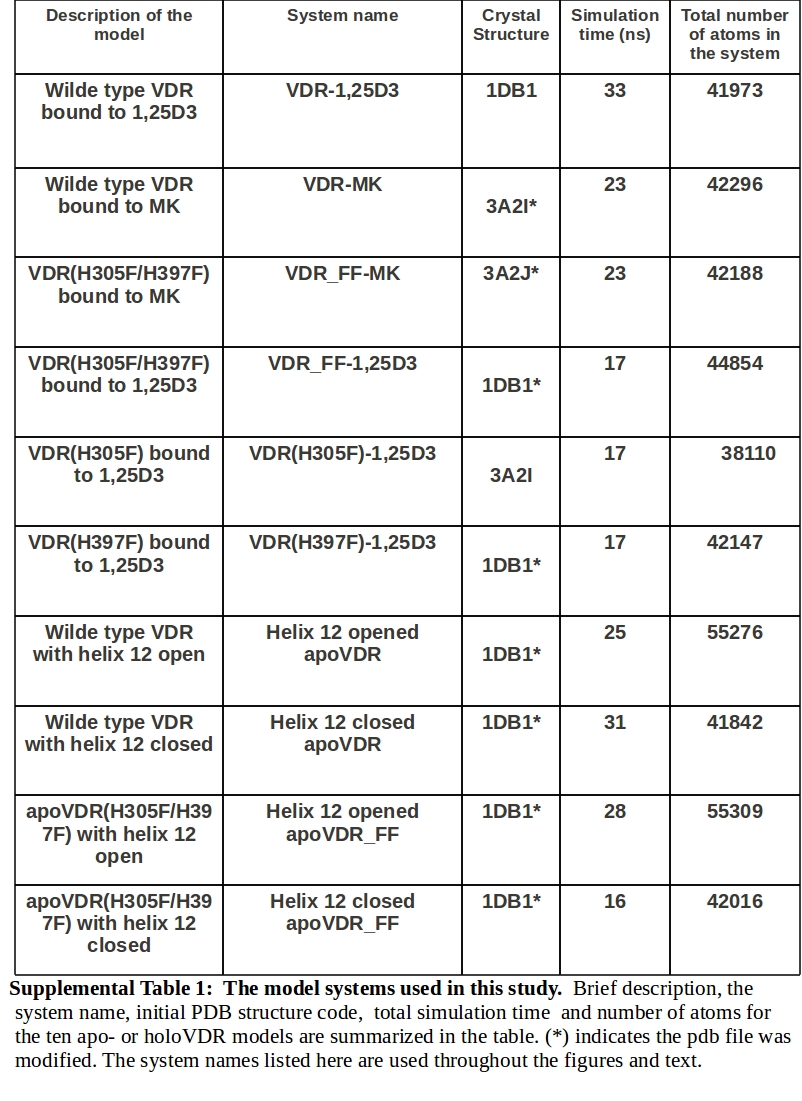

Supplement: Supplementary file 5 — Additional file 5: Figure S3. Rotameric states for the side-chains of ARG274 in VDR-1,25D. (a) 17ns run, (b) 32ns run, (c) 32ns run with crystal waters included. SC1, SC2, SC3 and SC4 are shown in magenta, yellow, green and blue respectively. (JPEG 347 KB) [file 40203_2012_2_MOESM5_ESM.jpeg]

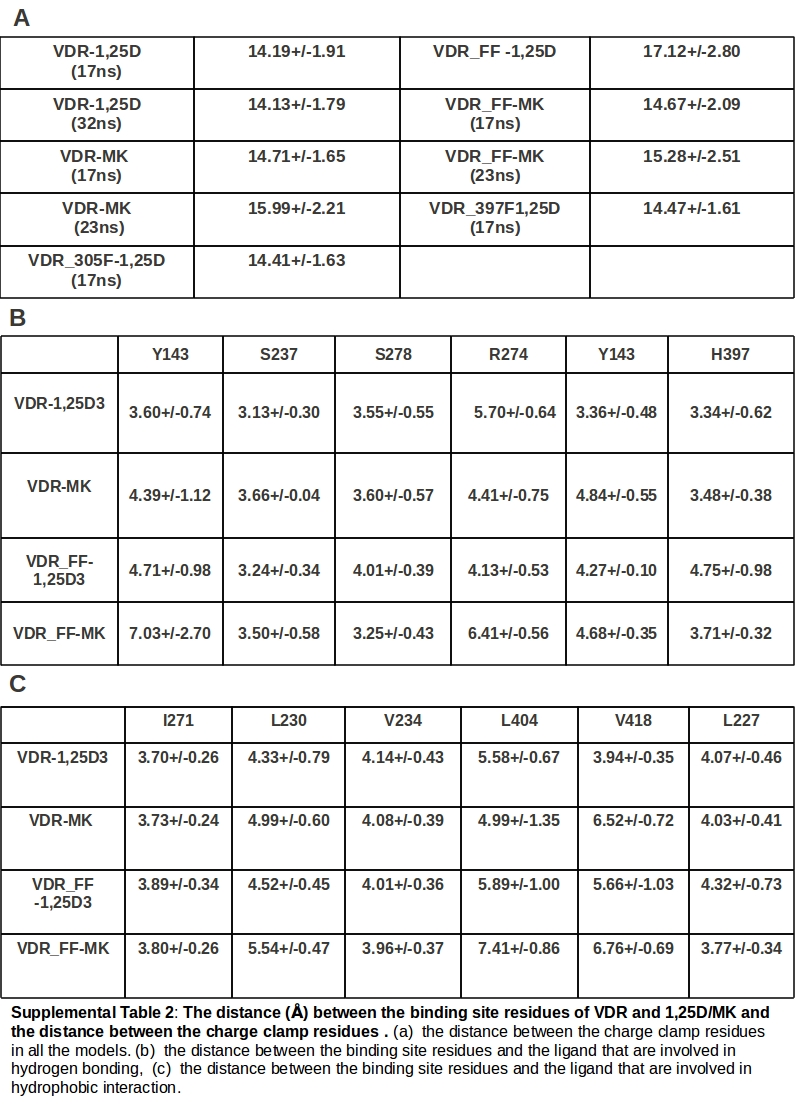

Supplement: Supplementary file 6 — Additional file 6: Figure S4. Rotameric state for side-chains of ARG274 in VDR-MK. (a) 17ns run, (b) 23ns run. SC1, SC2, SC3 and SC4 are shown in magenta, yellow, green, and blue respectively. The second side-chain of R274 in model a has a 10% occupancy at 180° and 2% occupancy at 90°, while in model b there is a 20% occupancy at 90°. (JPEG 357 KB) [file 40203_2012_2_MOESM6_ESM.jpeg]
